# “Flying Saucer”–Like Morphology in Infective Endocarditis Arising on a Stenotic Mitral Valve

**DOI:** 10.1016/j.jaccas.2026.106955

**Published:** 2026-02-12

**Authors:** Hiroki Iitsuka, Jun Yoshida, Akira Yoshii, Takanori Tsujimoto, Mayo Nakamura, Michihiro Yoshimura, Takashi Kunihara, Michifumi Tokuda

**Affiliations:** a Division of Cardiology, Department of Internal Medicine, The Jikei University School of Medicine, Minato, Japan; b Department of Cardiac Surgery, The Jikei University School of Medicine, Minato, Japan; c Department of Pathology, The Jikei University School of Medicine, Minato, Japan

**Keywords:** atypical morphology, degenerative mitral stenosis, mitral valve aneurysm, multimodal imaging, three-dimensional transesophageal echocardiography, vegetation

## Abstract

**Background:**

Infective endocarditis arising on a stenotic mitral valve is uncommon, and it may present with atypical morphology.

**Case Summary:**

We report a case of infective endocarditis in a patient with mild to moderate degenerative mitral stenosis who developed an extensive, broad-based vegetation nearly occluding the mitral valve, with an overlying contiguous aneurysm-like cystic structure. Transthoracic echocardiography demonstrated an elevated transmitral gradient, while transesophageal echocardiography revealed hemodynamic communication between the cystic structure and the left ventricle. Computed tomography showed contrast enhancement within the cavity. Three-dimensional transesophageal echocardiography was crucial in delineating the en face morphology and extent of the obstruction. The surgical and pathological findings confirmed a flat vegetation with pseudoaneurysmal features.

**Discussion:**

This high-risk morphology warrants urgent surgical consideration, as aneurysmal rupture could precipitate acute mitral regurgitation superimposed on obstruction.

**Take-Home Message:**

With the increasing prevalence of degenerative mitral stenosis, recognition of such morphologies is essential to guide timely surgical intervention.

## Visual Summary

**Figure:**
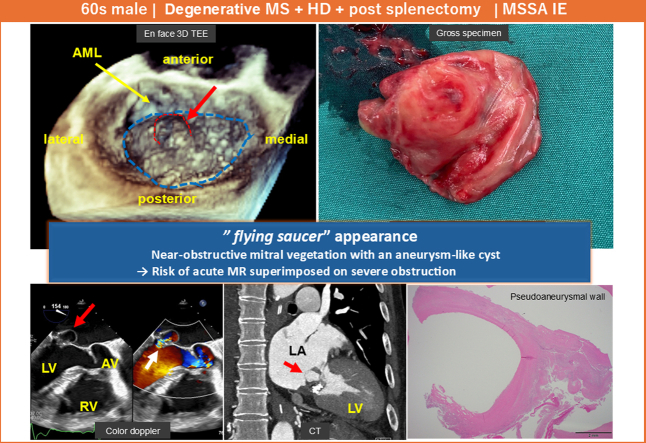

Degenerative mitral stenosis (MS) is becoming increasingly prevalent as the population ages. Although infective endocarditis (IE) arising on a stenotic mitral valve is relatively uncommon, it may present with distinctive morphologic features.

Here, we report a case of IE complicating degenerative MS that exhibited a characteristic disc-and-dome configuration, with important clinical implications.

## History of Presentation

A man in his 60s on chronic hemodialysis for 18 years owing to kidney failure secondary to nephrotic syndrome with renal infarction, complicated by recurrent vascular access occlusion requiring a graft shunt, presented with acute fever and altered mental status. He developed a fever of 39 °C the day before admission, followed by nausea, vomiting, and left hip pain. During dialysis, the patient became transiently unconscious and was transferred to the emergency department. On admission, his blood pressure was 95/71 mm Hg, heart rate 117 beats/min, respiratory rate 30 breaths/min, and body temperature 39.4 °C. Physical examination revealed erythema over the graft shunt in his left arm, conjunctival petechiae, Osler nodes, and an irregular diastolic murmur (Levine grade II) at the apex without pulmonary rales.

## Past Medical History

The patient's medical history included Evans syndrome treated with splenectomy 30 years prior, followed years later by antiphospholipid antibody syndrome, and long-standing atrial fibrillation. Two years prior, transthoracic echocardiography (TTE) demonstrated mild to moderate MS with leaflet thickening, posterior commissural fusion, and calcification (mean transmitral gradient: 5.6 mm Hg).

## Differential Diagnoses

The differential diagnoses for the source of infection included shunt and left hip abscess.

## Investigations

Laboratory studies showed leukocytosis (16,500/μL), elevated C-reactive protein level (51 mg/dL), and thrombocytopenia (55,000/μL). Coagulation studies revealed markedly prolonged activated partial thromboplastin time (143.5 seconds) and reduced prothrombin activity (24%), with elevated fibrin degradation products (15 μg/mL) and D-dimer levels (5.9 μg/mL). Fibrinogen levels were elevated (764 mg/dL). Although interpretation was complicated by underlying antiphospholipid antibody syndrome and chronic warfarin therapy, these findings, in conjunction with thrombocytopenia and systemic infection, were considered consistent with sepsis-associated coagulopathy with disseminated intravascular coagulation-like features.

**Figure 1:**
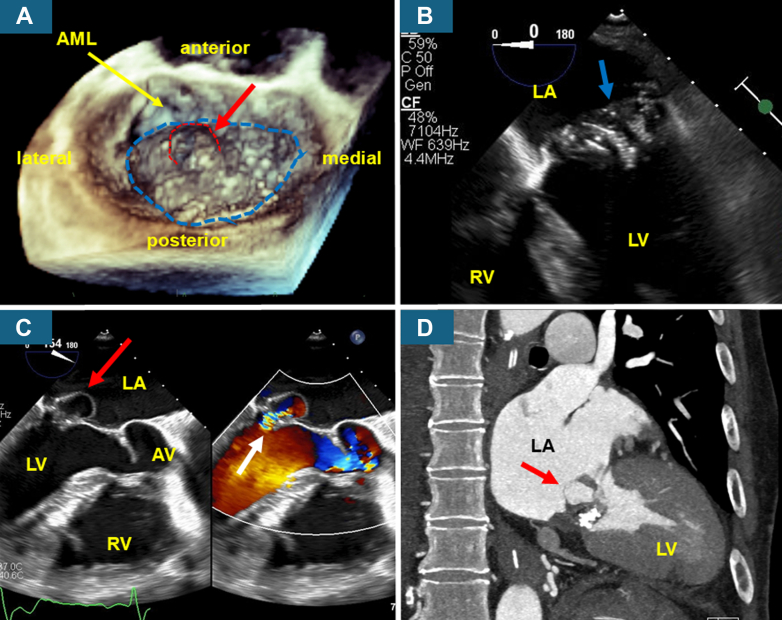
Multimodal Imaging Demonstrating Mitral Valve Vegetation With Aneurysm (A) Three-dimensional transesophageal echocardiography showing a broad, flat, disc-shaped vegetation (blue dashed line) with an overlying cystic structure (red arrow and red dashed line) covering the atrial surface of both the anterior and posterior mitral leaflet. (B) Two-dimensional transesophageal echocardiography midesophageal 4-chamber view revealed vegetation with substantial thickness deposited on and adherent to the mitral valve (blue arrow). (C) Color Doppler imaging long-axis view demonstrated systolic inflow (white arrow) from the left ventricular side into an associated cystic structure (red arrow), consistent with a mitral valve aneurysm. (D) Computed tomography revealed a cystic structure (red arrow) associated with the calcified mitral valve, showing contrast enhancement within the cavity. AML = anterior mitral leaflet; AV = aortic valve; LA = left atrium; LV = left ventricle; RV = right ventricle.

Contrast-enhanced computed tomography (CT) revealed a left hip abscess, while brain magnetic resonance imaging revealed an acute right parietal infarction with microbleeds. Blood cultures obtained on hospitalization day 1 grew Gram-positive cocci, later identified as methicillin-sensitive *Staphylococcus aureus* (MSSA). Doppler ultrasonography of the left arm graft shunt demonstrated a hypoechoic lesion surrounding the puncture site, suggestive of local infection. Cultures obtained from the graft shunt subsequently grew MSSA. In addition, imaging-guided aspiration of the fluid collection around the left hip joint yielded mildly purulent fluid, and cultures similarly grew MSSA. TTE revealed a heavily calcified mitral valve with a large, relatively immobile mass measuring 20 mm in diameter, resulting in severe MS (mean transmitral gradient: 12 mm Hg). The aortic valve was normal. These findings fulfilled the Duke criteria for definitive IE. Three-dimensional transesophageal echocardiography (TEE) on day 5 demonstrated a massive, broad-based vegetation covering approximately two-thirds of the atrial surface of the mitral leaflet, measuring 30.9 × 20.6 × 4.5 mm (Figure 1A, Video 1). Two-dimensional TEE revealed vegetation with substantial thickness deposited on and adherent to the mitral valve on midesophageal 4-chamber view (Figure 1B, Video 2). A cystic structure 15.3 × 14.9 × 11.4 mm was visualized on the vegetation, into which blood flow entered from the left ventricular side on color Doppler (Figure 1C, Video 3). CT also revealed a cystic structure associated with a calcified mitral valve, showing contrast enhancement within the cavity (Figure 1D). These findings suggested the formation of a mitral valve aneurysm (MVA). The aortic valve had a tricuspid morphology, and no vegetations or regurgitant jet were observed.

**Figure 2:**
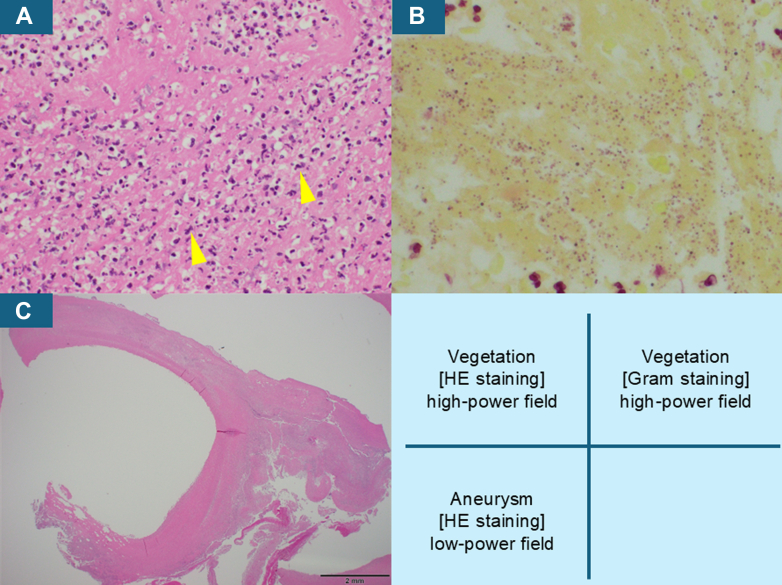
Histopathologic Examination of the Resected Specimen (A) The vegetation was composed of fibrin with inflammatory exudate predominantly consisting of neutrophils (arrowheads) (HE staining, high-power field). (B) Numerous Gram-positive cocci were also identified within the vegetation (Gram staining, high-power field). (C) The wall of the mitral valve aneurysm lacked the elastic fibers normally observed in the valve leaflets and was instead composed of fibrous connective tissue (HE staining, low-power field). HE = hematoxylin and eosin.

A histopathological examination of the resected specimen revealed that the vegetation was composed of fibrin with inflammatory exudate predominantly consisting of neutrophils contiguous with the valvular tissue (Figure 2A). Numerous Gram-positive cocci were also identified within the vegetation, confirming active bacterial infiltration (Figure 2B). The wall of the MVA lacked the elastic fibers normally observed in valve leaflets and was instead composed of fibrous connective tissue (Figure 2C), suggesting a pseudoaneurysmal structure rather than a true leaflet aneurysm.

## Management

Given the clinical context, the IE was presumed to have originated from either a graft shunt, left hip abscess, or both. Empirical therapy with meropenem, vancomycin, and cefazolin was initiated. In light of acute ischemic stroke with cerebral microbleeds and active native-valve IE, systemic anticoagulation was withheld because of the high risk of hemorrhagic transformation. Immediate valve surgery was deferred because the infected shunt graft and hip abscess represented uncontrolled sources, and early prosthetic implantation in the setting of disseminated infection was considered high risk. Therefore, the initial strategy prioritized source control.

**Figure 3:**
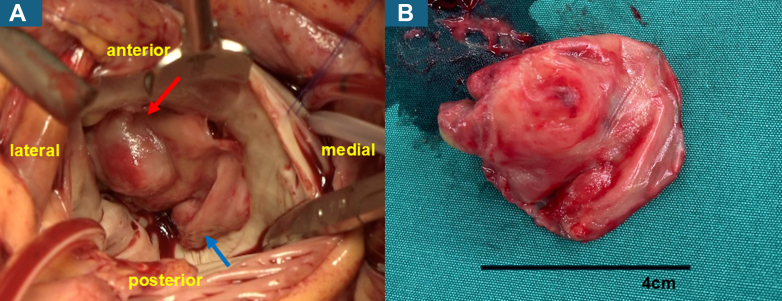
Intraoperative Findings and the Resected Specimen (A) Intraoperative findings demonstrated a vegetation overlying the mitral valve, characterized by flat vegetation (blue arrow) with a dome-like cystic cavity (red arrow) covering the leaflet. (B) Intraoperative macroscopic view of the excised vegetation showed a characteristic broad-based disc-like vegetation with a dome-shaped cystic component.

On day 3, the shunt graft was removed; however, the patient's fever persisted, leading to surgical debridement of the left hip abscess on day 5. Given the extent of valvular destruction and the poor likelihood of a medical response, the heart team decided to proceed with urgent surgery. Mitral valve replacement, tricuspid annuloplasty, and left atrial appendage resection were performed via full median sternotomy under cardiopulmonary bypass on day 6. Intraoperative inspection revealed thick, broad-based vegetation covering most of the mitral leaflet, with a cystic cavity consistent with an aneurysm (Figures 3A and 3B). The vegetation was confluent with the valve tissue, while areas of heavy calcification persisted and much of the native leaflet was destroyed.

## Outcome and Follow-Up

The postoperative course was uneventful. Thrombocytopenia during vancomycin and cefazolin therapy prompted a switch to cefepime; recurrent fever led to escalation to meropenem, and a total of 6 weeks of targeted intravenous antibiotic therapy was completed. At the 6-month follow-up examination, the patient remained clinically stable without any signs of infection recurrence.

## Discussion

The most distinctive feature of the present case is its highly unusual morphology. A thick vegetation extensively covered the mitral orifice and adhered firmly to the mitral leaflets in a broad-based, disc-like fashion, with a cystic structure forming on top, creating a characteristic “flying saucer”–like appearance. This configuration differs substantially from the more mobile, pedunculated vegetations typically observed in IE associated with mitral regurgitation (MR).

Although the patient had pre-existing mild to moderate degenerative MS, IE is generally considered less common in MS than in MR.^1^ In this case however, multiple predisposing factors likely contributed to the development of IE, including chronic hemodialysis and prior splenectomy, both of which represent immunocompromised conditions. In addition, infection with MSSA, a highly tissue-invasive pathogen, likely promoted extensive valvular destruction.

A previous report described functional obstruction of the mitral orifice caused by a large vegetation in the setting of mild MS due to mitral annular calcification; the investigators speculated that blood flow stasis (similar to the mechanism of thrombus formation) facilitated the development of large vegetations.^2^ Although blood flow stasis and reduced leaflet mobility are both consequences of MS, they represent distinct pathophysiologic contributors: Flow stasis may facilitate the volumetric growth of vegetation, whereas restricted leaflet motion may determine its broad-based, flattened pattern of attachment. In the present case, reduced leaflet mobility likely allowed bacterial adherence and accumulation on the leaflet surface, resulting in an extensive, flat vegetation firmly adherent to the mitral valve.

Importantly, the present case further demonstrated formation of a cystic structure contiguous with the vegetation. Once bacterial seeding weakens the leaflet tissue, continuous exposure to left ventricular pressure may promote cystic expansion, resulting in an aneurysm-like (pseudoaneurysmal) structure. This pathophysiologic interplay provides a plausible explanation for the unusual disc-and-dome configuration observed in our patient.

MVA most commonly develops as a complication of aortic valve IE^1^ and has also been reported in association with mitral valve prolapse.^3^ To our knowledge however, there have been no prior reports describing an aneurysm-like structure of the mitral valve complicating IE in the setting of MS. The pathological examination in the present case demonstrated loss of elastic fibers within the cystic wall, suggesting a prerupture state.

Clinically, this morphology represents a high-risk condition. The vegetation already caused severe MS by nearly occluding the mitral orifice, and rupture of the cystic structure would likely have resulted in superimposed acute MR.^3^ The simultaneous occurrence of severe MS and acute MR is associated with catastrophic hemodynamic collapse. Accordingly, the presence of a broad-based obstructive vegetation with a cystic component should be regarded as a critical warning sign that strongly supports urgent surgical intervention.

Multimodality imaging played a pivotal role in the diagnosis and management of this case. TEE demonstrated hemodynamic communication between the cystic structure and the left ventricle, while CT confirmed contrast enhancement within the cavity. Such communication is considered a key mechanism in the development of aneurysm-like or pseudoaneurysmal lesions in IE. In addition, CT is particularly useful for identifying perivalvular complications such as abscess, fistula, or dehiscence,^4^ none of which were observed in this case. Three-dimensional TEE, in particular, provided an en face view of the mitral valve, clearly demonstrating extensive coverage of the mitral orifice and resultant inflow obstruction. Although the mean transmitral gradient measured by TTE is useful for estimating the severity of MS, it is influenced by heart rate, cardiac output, and the presence of MR.^5^ Further, it may not adequately reflect the spatial extent of obstruction or associated cystic communication. Therefore, comprehensive assessment using complementary imaging modalities is essential.

In developed countries, the prevalence of rheumatic MS has declined, whereas degenerative MS associated with mitral annular calcification is increasing with an aging population.^6^ In parallel, *S* *aureus* has become an increasingly common cause of IE, particularly in health care–associated infections.^7^ The present case, characterized by degenerative MS complicated by *S* *aureus* IE in an immunocompromised host, reflects these contemporary epidemiologic trends. As the incidence of IE arising on degenerative MS is expected to increase, recognition of characteristic high-risk morphologies such as the present appearance is essential for its timely diagnosis and prompt intervention in daily clinical practice.

**Visual Summary:**
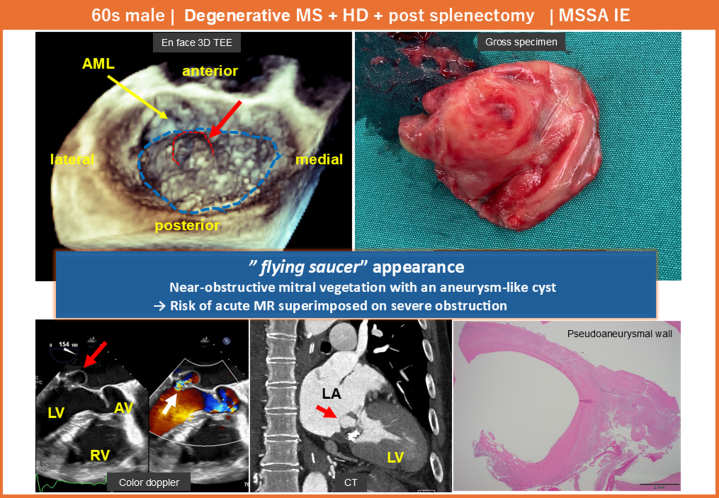
Infective Endocarditis Arising on Degenerative Mitral Stenosis With a Characteristic “Flying Saucer”–Like Morphology A broad-based vegetation nearly occluded the mitral orifice and was accompanied by a contiguous aneurysm-like cystic structure with left ventricular communication, representing a high-risk configuration that warranted urgent surgical intervention. AML = anterior mitral leaflet; AV = aortic valve; HD = heart disease; LA = left atrium; LV = left ventricle; MS = mitral stenosis; RV = right ventricle.

## Conclusions

This case illustrates a rare and high-risk manifestation of IE arising on a stenotic mitral valve, characterized by an extensive, broad-based vegetation with a contiguous aneurysm-like structure forming a distinctive disc-and-dome configuration. Such morphology represents a critical warning sign that warrants urgent surgical consideration and highlights the essential role of multimodality imaging in clinical decision-making.

## Funding Support and Author Disclosures

The authors have reported that they have no relationships relevant to the contents of this paper to disclose.

**Take-Home Messages**

• Infective endocarditis arising on a stenotic mitral valve can present with a distinctive morphology characterized by an extensive flat vegetation with a contiguous aneurysm-like cystic structure (“flying saucer” appearance), representing a high-risk phenotype associated with mitral inflow obstruction and potential rupture leading to acute mitral regurgitation and catastrophic hemodynamic collapse.
• As the prevalence of degenerative mitral stenosis continues to increase, recognition of such characteristic and high-risk morphologies is increasingly important to facilitate timely diagnosis and appropriate surgical intervention.

## Appendix

Video 1 Three-Dimensional Transesophageal Echocardiography (En Face View of the Mitral Valve) Three-dimensional transesophageal echocardiography demonstrated a mitral valve aneurysm within the vegetation of the mitral valve, bulging during systole and partially collapsing during diastole.

Video 2 Transesophageal Echocardiography (Midesophageal 4-Chamber View) Two-dimensional transesophageal echocardiography midesophageal 4-chamber view revealed vegetation with substantial thickness deposited on and adherent to the mitral valve.

Video 3 Color Doppler Transesophageal Echocardiography (Long-Axis View of the Mitral Valve) Two-dimensional long-axis transesophageal echocardiography demonstrated systolic inflow from the left ventricle into the mitral valve aneurysm, and diastolic outflow from the aneurysm back into the left ventricle.
